# Numerical and Experimental Research on Identifying a Delamination in Ballastless Slab Track

**DOI:** 10.3390/ma12111788

**Published:** 2019-06-02

**Authors:** Guopeng Fan, Haiyan Zhang, Wenfa Zhu, Hui Zhang, Xiaodong Chai

**Affiliations:** 1 Shanghai Institute for Advanced Communication and Data Science, School of Communication and Information Engineering, Shanghai University, Shanghai 200444, China; phdfanry@shu.edu.cn (G.F.); zh6154@126.com (H.Z.); 2 School of Urban Railway Transportation, Shanghai University of Engineering Science, Shanghai 201620, China; zhuwenfa1986@163.com (W.Z.); xdchai@sues.edu.cn (X.C.)

**Keywords:** wavenumber method, delamination, ballastless slab track, shear waves, removing the surface wave, resolution

## Abstract

This paper aims to adopt the total focusing method (TFM) and wavenumber method for characterizing a delamination in ballastless slab track. Twelve dry point contact (DPC) transducers located at the upper surface of the slab track compose a linear array. These transducers are employed to actuate shear waves, which are suitable for identifying the delamination. The technique of removing the surface wave has been implemented for only retaining the scattered wave caused by the delamination and the reflected wave from the bottom of bed plate. Numerical and experimental results demonstrate that the delamination and bottom of the bed plate can be identified by the proposed methods. Furthermore, the near-surface pseudomorphism is distinctly restrained after removing the surface wave. Compared to TFM, the wavenumber method has the great advantages of improving computational performance and lateral resolution. However, they have no significant difference in the longitudinal resolution. Furthermore, it has been confirmed that the lateral resolution can be affected by the amount of transducers. This paper can provide valuable suggestions on improving the computational performance and the imaging accuracy when we identify a delamination in ballastless slab track.

## 1. Introduction

The ballastless track has been widely applied in high-speed railways [1]. Compared to the conventional ballast track, the ballastless track has the advantages of high structural stability, good stiffness uniformity, long service life, and less maintenance work [2,3]. Nevertheless, the complex natural environment, rain erosion, train load, and thermal load may lead to the ballastless track bearing some structural diseases, such as cracks, air voids, and delaminations [4,5,6]. These diseases may not be easily recognized by visual investigation.

Among these diseases, the delamination located between multi-layers is frequently observed in ballastless track. The dynamic behavior of slab track might be affected by the smaller delamination. However, the larger delamination will dramatically reduce the safety performance, and even result in the potential failure of slab track, such as fracture and transfiguration. It is significant to obtain the location and depth of the delamination. Many researchers have proposed various nondestructive testing (NDT) techniques, such as ground penetrating radar (GPR) [7,8], active infrared thermography (IRT) technique [9,10], and ultrasonic testing (UT) method [11].

Hong et al. utilized high frequency GPR system for periodically monitoring reinforcement corrosion in concrete, and visualizing the accumulative corrosion progress [12]. However, the identification of void diseases in a ballastless track using GPR is difficult. This is mainly due to the fact that the echoes caused by the void will be affected by the reflected wave occurring on the rebars surface. Void diseases are especially located at the lower part of rebars.

Tran et al. proposed the square pulse thermography (SPT) to identify the subsurface delamination in concrete [13]. They investigated the effect that steel bars exerted on the detectability of delamination. The results showed that the absolute contrast of a delamination was reduced slightly under the effect of steel bars. In addition, the test result was relevant to the width-to-depth ratio of the delamination. Similarly, Huh et al. applied long pulsed thermography (LPT) to characterize the delamination in a concrete slab [14]. They employed the non-dimensional prefactor *k* to predict the depth of a delamination and evaluated the effects of steel bars. The active infrared thermography was an effective tool for detecting the delamination with the lower water/cement ratio. It was verified that the delamination with a large size or being located near the surface was more easily detected by using IRT [15]. However, IRT is unsuited for detecting the deep delamination. Moreover, IRT is strongly influenced by environmental conditions.

The UT method is a commonly used NDT technique and has shown great potential for structural health monitoring (SHM). Damage imaging methods based on elastic waves are multifarious, such as synthetic aperture focusing technique (SAFT) [16,17], total focusing method (TFM) [18], ultrasonic phased array [19], reverse time migration (RTM) [20], and ultrasonic tomography [21].

It is well known that the high frequency-thickness product leads to multimodal guided waves coexisting [22]. Under this condition, the mode identification and separation will become difficult. As a result, it is not easy to identify the damage existing in multi-layers concrete structure using guided waves. Li et al. adopted guided waves to evaluate the integrity of CFRP (Carbon Fiber Reinforced Polymer)-reinforced concrete structures [23]. Their work indicated that debonding indices were only applicable for quantifying the severity of debonding. Song et al. demonstrated that interface bonding conditions of steel–clad concrete structures could be quantitatively evaluated using attenuation characteristics of guided waves [24].

Considering the above reasons, many researchers tend to apply bulk waves for detecting damage in concrete structure. Schabowicz found that the ultrasonic tomography technique can not only be used to determine the location and size of a defect, but also to estimate the thickness of a concrete member [25]. One minor flaw was that the components of the received signals had not yet been analyzed. Beniwal et al. investigated the ultrasonic imaging of concrete using scattered elastic wave modes [26]. Compared with the traditional TFM, the weighted sum technique and statistical technique were conducive to improve the detection performance. However, the near-surface pseudomorphism could not be ignored as the surface wave with high amplitude was contained in the received signal. 

For the purpose of maintaining the safety and stability of the high-speed railway, the TFM and wavenumber method based on full matrix capture (FMC) were used for identifying a delamination in ballastless slab track. In this paper, 12 DPC transducers located at the upper surface of the slab track were employed to actuate shear waves [27]. The main work was focused on suppressing the near-surface pseudomorphism, improving the computational performance, and retaining a relatively favorable accuracy. 

This paper is organized with seven sections, including this Introduction. Section 2 illustrates the theory of TFM and the wavenumber method. Section 3 presents the numerical results. Experimental results are explicated in Section 4. Section 5 concludes the discussion. Section 6 summarizes the main work. Section 7 provides the recommendation for future research. 

## 2. Principles of the TFM and the Wavenumber Method

### 2.1. Sound Field Transmission and Reception

**Figure 1:**
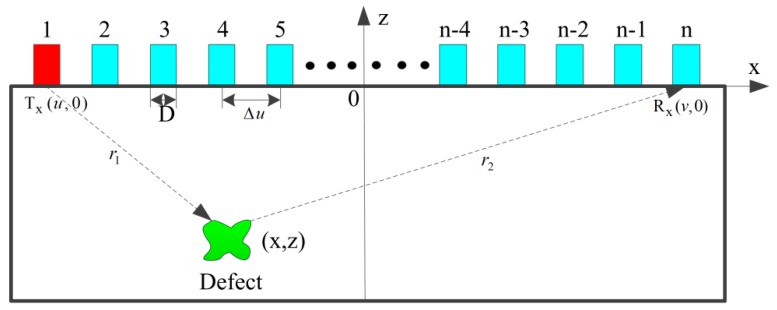
Transmission and reception of the ultrasonic phased array.

The transmission and reception of the ultrasonic phased array is introduced based on the two-dimensional (2-D) medium. The schematic diagram used for describing the process of sound field transmission and reception is shown in Figure 1. Linear array elements are located at the upper surface of the material, while a defect is embedded into the material. The X axis is parallel to the material’s surface, and the Z axis is perpendicular to the material’s surface. The elements are spaced at a fixed pitch Δu, and each element has a width of D. The coordinate origin is at the center of the array. 

**Figure 2:**
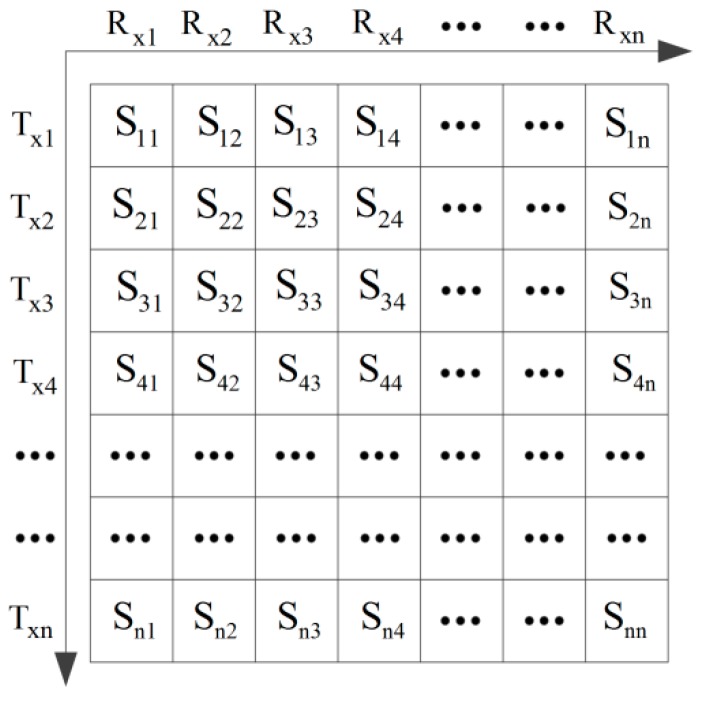
Schematic diagram of full matrix capture.

Generally, the signals for each transmitter–receiver pair within the array are recorded in space and time. The full matrix capture data [28] is shown in Figure 2. $S_{ij}$ denotes the signal which is obtained under transmitter element i and receiver element j. The three-dimensional (3-D) matrix corresponds to the transmitter and receiver positions and the temporal axis. (u,0) and (v,0) are the Cartesian coordinates of the transmitter and receiver elements. (x,z) is the Cartesian coordinate of the defect. $r_{1}$ is the distance between the transmit element and the defect, and $r_{2}$ is the distance between the receive element and the defect. Naturally, $r_{1}$ and $r_{2}$ can be expressed as:

(1) $$r_{1}=\sqrt{{\left(x-u\right)}^{2}+z^{2}}$$

(2) $$r_{2}=\sqrt{{\left(x-v\right)}^{2}+z^{2}}$$

### 2.2. Principle of the TFM

TFM is a type of ultrasonic imaging and it has been widely used for Non-destructive Evaluation (NDE). The first step of implementing TFM algorithm is discretizing the target region into a grid. Then, the travelled time is calculated by the travelled distance and the group velocity [29]. This operation can be denoted as:

(3) $$T_{ij}\left(x,z\right)=\frac{\sqrt{{\left(x-x_{i}\right)}^{2}+{\left(z-z_{i}\right)}^{2}}+\sqrt{{\left(x-x_{j}\right)}^{2}+{\left(z-z_{j}\right)}^{2}}}{C_{g}}$$

where, $\left(x_{i},z_{i}\right)$ is the Cartesian coordinates of the transmitting sensor. $\left(x_{j},z_{j}\right)$ is the Cartesian coordinates of the receiving sensor (x,z) is the Cartesian coordinates of the gird. $c_{g}$ is the group velocity. $T_{ij}(x,z)$ denotes the travelled time.

Finally, the amplitude of the signal’s envelope (relative to the transducer pair) associated to this travelled time is extracted and added to the pixel intensity [30]. This operation is repeated for each transmitter-receiver pair.

(4) $$I\left(x,z\right)=\left|\sum_{i=1}^{N}\sum_{j=1}^{N}S_{ij}\left(T_{ij}\left(x,z\right)\right)\right|$$

here, I(x,z) is the intensity of the pixel located at the Cartesian coordinates (x,z). *N* denotes the amount of sensors. $S_{ij}$ is the amplitude of the signal’s envelope.

### 2.3. Principle of the Wavenumber Method

The wavenumber method based on FMC is adapted from the theories described by Hunter et al. and Moghimirad et al. [31,32]. The algorithm is expatiated as follows. First of all, the received echo signal e(t,u,v) is given by E(ω,u,v) in frequency domain.

(5) E(ω,u,υ)=P(ω)∬f(x,z)g(ω,x−u,z)g(ω,x−v,z)dxdz

where P(ω) is the Fourier transform of the transmitted signal P(t).f(x,z) denotes a continuous distribution of the scatterer. The 2-D free-space Green’s function can be expressed as [33]:

(6) $$g(\omega ,x,z)=\frac{-j}{4\pi }\int \frac{\exp(-j|z|\sqrt{k^{2}-k_{x}^{2}}+jk_{x}x)}{\sqrt{k^{2}-k_{x}^{2}}}dk_{x}$$

Naturally, the temporal spectrum of the received echo signal can be rewritten as:

(7) $$\begin{array}{c} E(\omega ,u,v)=P(\omega )\frac{-1}{{\left(4\pi \right)}^{2}}\iint \frac{\exp(jk_{u}u+jk_{v}v)}{\sqrt{k^{2}-k_{u}^{2}}\sqrt{k^{2}-k_{v}^{2}}}[\iint f(x,z)\times \exp(-j(k_{u}+k_{v})x\\ -j(\sqrt{k^{2}-k_{u}^{2}}+\sqrt{k^{2}-k_{v}^{2}})z)\times dxdz]dk_{u}dk_{v} \end{array}$$

The next step is to obtain the frequency-wavenumber spectrum $E(\omega ,k_{u},k_{v})$ by applying the 2-D fast Fourier transform [34] of E(ω,u,v).

(8) $$E(\omega ,k_{u},k_{v})=P(\omega )\frac{-F(k_{u}+k_{v},\sqrt{k^{2}-k_{u}^{2}}+\sqrt{k^{2}-k_{v}^{2}})}{{\left(4\pi \right)}^{2}\sqrt{k^{2}-k_{u}^{2}}\sqrt{k^{2}-k_{v}^{2}}}$$

here, F is the spatial Fourier transform of the scattering function f(x,z). The process of transforming the E(ω,u,v) to $E(\omega ,k_{u},k_{v})$ can also be accomplished by using the 3-D fast Fourier transform [35]. The system model of the incident wavenumber $k_{u}$ is given by:

(9) $$F(k_{x},k_{z}|k_{u})=-{\left(4\pi \right)}^{2}S^{-1}\{\sqrt{k^{2}-k_{u}^{2}}\sqrt{k^{2}-k_{v}^{2}}E(\omega ,k_{v}|k_{u})\}$$

where the operator $S^{-1}\{\}$ denotes the Stolt mapping, which can be obtained from the Equations (10)–(12). $E(\omega ,k_{v}|k_{u})$ denotes the 2-D slices for each value of $k_{u}$. In this case, the system model in wavenumber domain is obtained by summing together $F(k_{x},k_{z}|k_{u})$. The operation given by Equation (13) is for the purpose of reducing the side lobes.

(10) $$k_{x}=k_{u}+k_{v}$$

(11) $$k_{z}=\sqrt{k^{2}-k_{u}^{2}}+\sqrt{k^{2}-k_{v}^{2}}$$

(12) $$k=\frac{\pm \sqrt{k_{z}{}^{4}+2(k_{u}{}^{2}+{\left(k_{x}-k_{u}\right)}^{2})k_{z}{}^{2}+k_{u}{}^{4}+{\left(k_{x}-k_{u}\right)}^{4}-2k_{u}{}^{2}{\left(k_{x}-k_{u}\right)}^{2}}}{2k_{z}}$$

(13) $$\hat{F}(k_{x},k_{z})=\int F(k_{x},k_{z}|k_{u})dk_{u}$$

**Figure 3:**
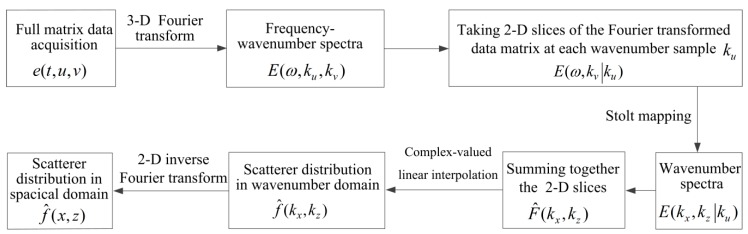
Flow chart of the wavenumber method based on the full matrix capture.

Finally, the image of the scatterer distribution can be reconstructed by applying the 2-D inverse Fourier transform (IFFT2) and this operation is given by Equation (14). It is important to note that IFFT2 is based on the uniform baseband grid. However, the raw baseband grid is inhomogeneous as the Stolt mapping is a kind of non-linear coordinate transforms [36,37]. Therefore, $\hat{f}(k_{x},k_{z})$ is obtained by implementing the complex-valued linear interpolation on $\hat{F}(k_{x},k_{z})$. The wavenumber method based on FMC is summarized and its flow chart is shown in Figure 3.

(14) $$\hat{f}(x,z)=\frac{1}{{\left(2\pi \right)}^{2}}\iint \hat{f}(k_{x},k_{z})\exp(jk_{x}x+jk_{z}z)dk_{x}dk_{z}$$

## 3. Numerical Simulation

### 3.1. Finite Element Model

A commercial software package called PZFlex was used to create the 2-D finite element model. PZFlex is the time domain finite element method (FEM). The complex acoustic wave propagation can be simulated using this method [38]. 

**Figure 4:**
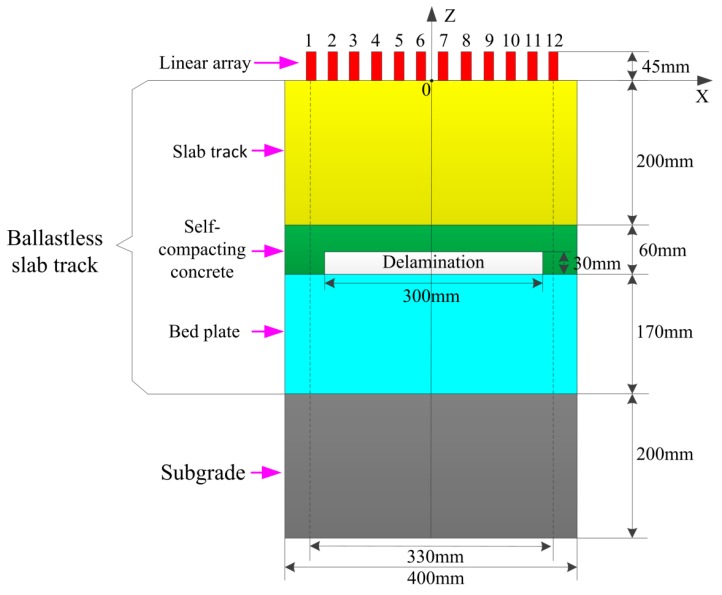
Finite element model.

The ballastless slab track is a typical concrete structure with three layers. It consists of the track slab, the self-compacting concrete and the bed plate from top to bottom. It should not be ignored that the subgrade is under the bed plate. The cross-section of the ballastless slab track in longitudinal direction is shown in Figure 4. Parameters of the finite element model are listed in Table 1. The upper and lower interfaces of the finite element model are with the free boundary conditions, while the left and right interfaces are with the absorbing boundary conditions. The sizes of the slab track, the self-compacting concrete, the bed plate and the subgrade are 400 mm × 200 mm, 400 mm × 60 mm, 400 mm × 170 mm, and 400 mm × 200 mm, respectively. The delamination with the size of 300 mm × 30 mm is located at the boundary between the self-compacting concrete and the bed plate. Twelve transducers are located at the upper surface of the slab track, and the central distance between adjacent transducers is 30 mm. The vibration direction of the transducer is along the horizontal direction, which is suitable to actuate the shear wave. The parameters of the ultrasonic transducer array are listed in Table 2.

**Figure 5:**
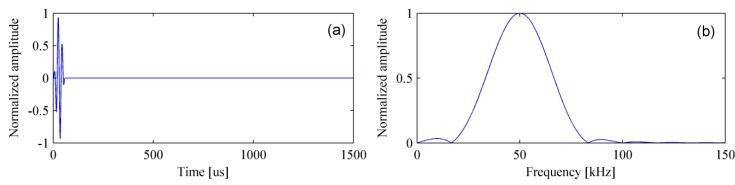
The excitation signal in the (**a**) time-domain and (**b**) frequency-domain.

Considering that the damping existed in slab track can not be ignored under the higher frequency, the central frequency of the excitation source is set at 50 kHz. Figure 5 shows the excitation signal, which is a three-cycle sinusoidal tone burst modulated by the Hanning window. In our test, the shear wave was about 2,466 m/s. Therefore, the wavelength was 49.320 mm (λ = 2466/50 = 49.320 mm). It is conducive to the detection of delamination when the value of wavelength remains in a reasonable range. The sampling interval was only 1 mm (λ/50). Apparently, the sampling interval satisfies the sampling theorem and can provide a good spatial resolution. 

The process of the excitation and reception should be in accordance with the FMC. In other words, each transducer has a chance to act as the excitation, and the acoustic signals are received by all the transducers. Naturally, a total of 144 signals will be received as the linear array consists of twelve transducers.

### 3.2. FEM Results

#### 3.2.1. Snapshots of the Wavefield

**Figure 6:**
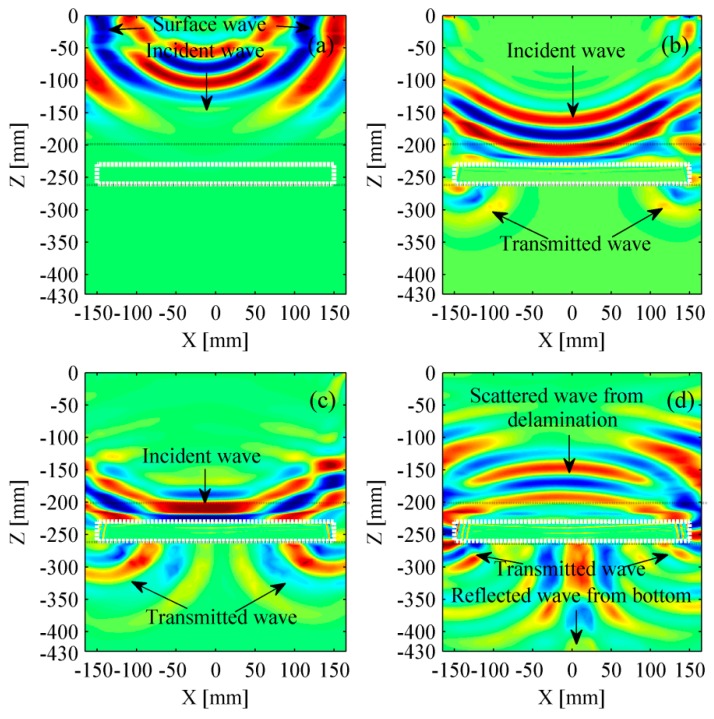
Snapshots of wavefield. (**a**) 66 μs; (**b**) 107 μs; (**c**) 127 μs; and (**d**) 152 μs. The rectangular area delimited by the white dotted lines denotes the delamination. The rectangular area delimited by the black dotted lines represents the self-compacting concrete.

The interactions between wavefield and the delamination have been analyzed by many researchers. Lin et al. described a layered structure with an embedded delamination and illustrated the screenshots of the wave propagation [39]. The reflection and refraction seemed complex at the overlay-specimen interface as the wave propagation velocities were obviously different in multi-layers structure. Figure 6 shows the snapshots of the ballastless slab track in longitudinal direction. These snapshots are generated by the FEM program when the sixth transducer acts as the excitation source. It should be noted that the wavefield in the subgrade has not been shown as we focus on the ballastless slab track. The incident, reflected, and transmitted waves can be observed. Figure 6d indicates that the scattered wave from the delamination is clear while the reflected wave from the bottom is very weak.

#### 3.2.2. FEM Results

**Figure 7:**
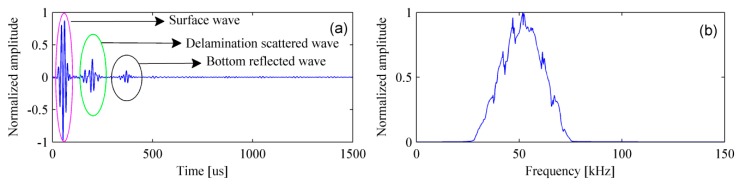
The simulated signal in the (**a**) time-domain and (**b**) frequency-domain.

**Figure 8:**
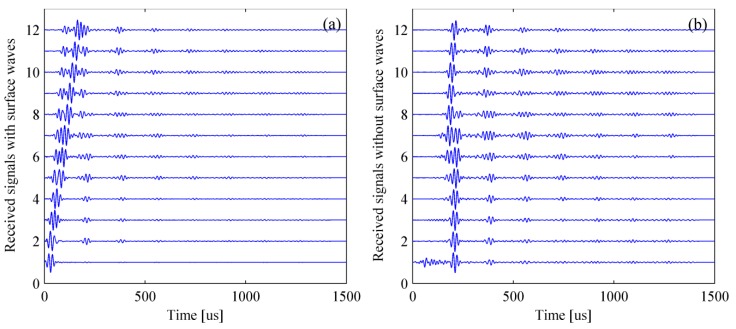
Removing surface wave for multiple signals. (**a**) Simulated signals with surface waves and (**b**) simulated signals without surface waves.

Figure 7 shows the simulated signal in the time- and frequency-domain. It can be clearly found that the amplitude of surface wave is much greater than that of the scattered wave caused by the delamination and the reflected wave from bottom of the bed plate. In fact, it is only necessary to retain the scattered and reflected waves as they are beneficial to the detection. A normal method is to subtract the signal acquired in the pristine structure from that acquired in the damaged structure. The process of removing surface wave for multiple signals is shown in Figure 8.

**Figure 9:**
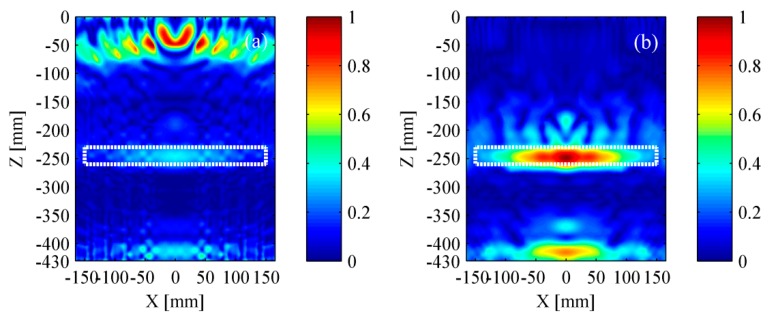
Simulation results based on the TFM (total focusing method). (**a**) Result with surface wave and (**b**) result without surface wave. The rectangle with white dotted line represents the location of a delamination.

The simulation results based on TFM are shown in Figure 9. The rectangle with white dotted line represents the location of delamination. The delamination and the bottom of bed plate both were obscure in Figure 9a as the pseudomorphism caused by the surface wave was conspicuous. It could be found that the near-surface pseudomorphism was distinctly suppressed after removing the surface wave. Thus, the delamination and the bottom of bed plate were clearly seen in Figure 9b. As a whole, the location of identified delamination matches well with its actual location. However, the identified delamination is shorter than its actual size, revealing that the lateral resolution of the TFM must be improved.

**Figure 10:**
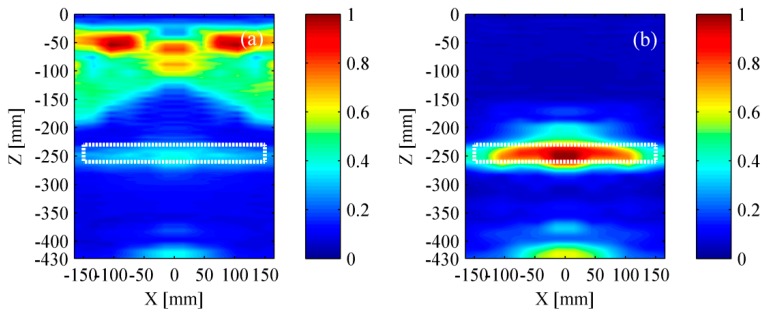
Simulation results based on the wavenumber method. (**a**) Result with surface wave and (**b**) result without surface wave. The rectangle with white dotted line represents the location of a delamination.

The simulation results based on wavenumber method are shown in Figure 10. Benefiting from removing the surface wave, the delamination and the bottom of bed plate were well identified. Furthermore, the identified delamination area matched well with its actual size. Compared to TFM, the wavenumber method took less time in obtaining the intensity imagery while maintaining a relatively accurate location and better lateral resolution.

## 4. Experimental Verification

### 4.1. Experimental Setup

**Figure 11:**
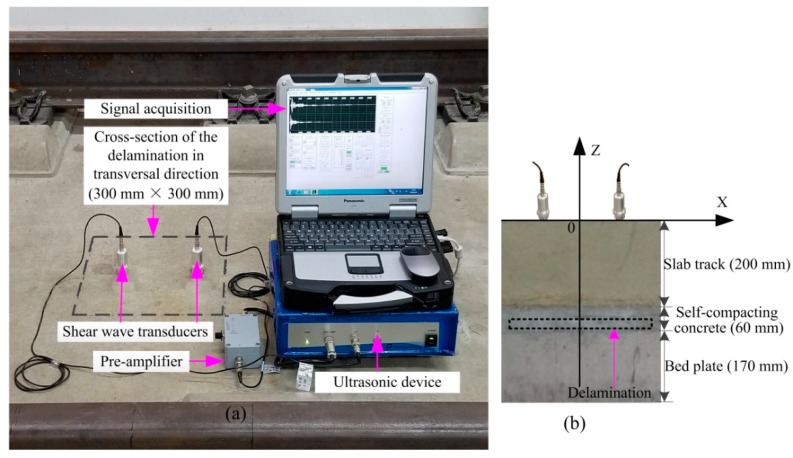
Experimental setup. (**a**) Cross-section of the delamination in transversal direction and (**b**) cross-section of the delamination in longitudinal direction.

The experimental system is composed of DPC transducers, an ultrasonic device (JPR-600C, Japan Probe, Yokohama, Japan), a pre-amplifier (PR-60A-06, Japan Probe, Yokohama, Japan) and the signal acquisition interface. The DPC transducer is a piezoelectric ceramic-based transducer with the central frequency of 50 kHz, and its bandwidth is about ±60% of the central frequency. The pre-amplifier was used to improve the signal-to-noise ratio (SNR) and its gain was set at 60 dB. The sampling frequency was set at 1 MHz. It is a suitable time to record the signal when the waveform tends to be stable. Each measurement was repeated 15 times in order to reduce the measurement error. The signal acquisition interface can display the detailed information of received signals, such as the sample interval, trigger point, amplitude, etc. The experimental conditions were in agreement with the simulation conditions. In fact, the ultrasonic device only has two channels. Nevertheless, the FMC still can be successfully completed. More specifically, one channel was used for transmitting. The other one was used for picking up direct and reflected waves. First of all, the excitation source was located at the fixed position. Then, the receiver was removed with a fixed interval after the signal was recorded. Thus, multi-channel received signals could be successfully obtained by using two transducers. The experimental setup is shown in Figure 11.

### 4.2. Experimental Results

#### 4.2.1. Removing Surface Wave

**Figure 12:**
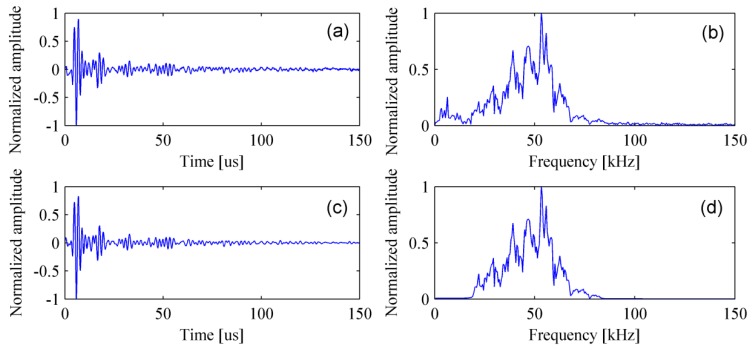
Band-pass filtering of an experimental signal. (**a**) Original signal; (**b**) spectrum of the original signal; (**c**) filtered signal; and (**d**) spectrum of the filtered signal.

Filtering and removing surface wave are required for experimental signals. First of all, the received signals are filtered by a Chebyshev band-pass filter [40] with a bandwidth of 20–80 kHz. Consequently, the noise is successfully removed and the filtered signal is shown in Figure 12.

**Figure 13:**
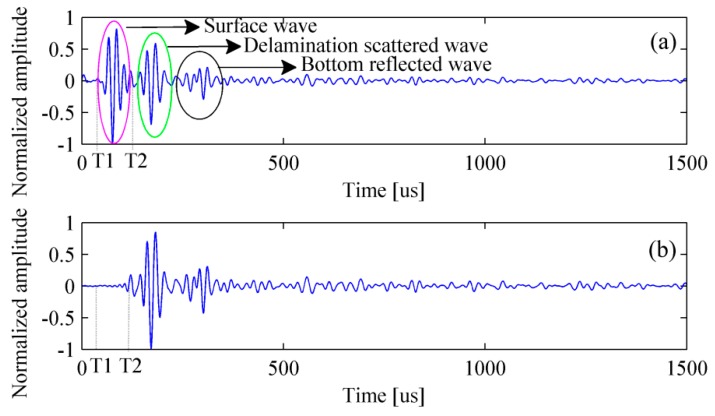
Removing surface wave for a single signal. (**a**) Experimental signal with surface wave and (**b**) experimental signal without surface wave.

**Figure 14:**
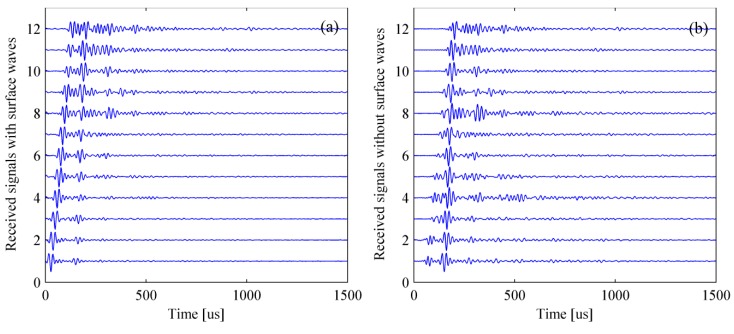
Removing surface wave for multiple signals. (**a**) Experimental signals with surface waves and (**b**) experimental signals without surface waves.

Generally, it is very hard to obtain the same surface wave in the pristine structure and the damaged structure as the real ballastless slab track is nonhomogeneous. Therefore, the method of removing surface wave is different from the case of FEM. In our test, the surface wave, the scattered wave from the delamination, and the reflected wave from the bottom were segregative in the time-domain. Therefore, the surface wave can be separated in the time-domain. The surface wave velocity of the track slab denoted by $C_{r}$ can be obtained by calculating the following equation [41]:

(15) $$C_{r}=\frac{0.87+1.12u}{1+u}\sqrt{\frac{E}{\rho }\left(\frac{1}{2(1+u)}\right)}$$

where, u is the Poisson’s ratio. ρ is the density. E is elasticity modulus. The values of these parameters have been listed in Table 1. The surface wave velocity of the slab track is about 2249 m/s. The travelled time of surface wave $T_{1}$ can be written as:

(16) $$T_{1}=\frac{\sqrt{{\left(x_{j}-x_{i}\right)}^{2}+{\left(z_{j}-z_{i}\right)}^{2}}}{C_{r}}$$

(17) $$T_{2}=T_{1}+T_{0}$$

where, $T_{0}$ is the duration of the original excitation signal envelope. $T_{2}$ is the deadline of the surface wave. The received signal is multiplied by a very small ratio coefficient before $T_{2}$ and filtered in the frequency-domain. In this paper, the process of removing surface wave is illustrated under the condition that No.1 transducer is selected as the excitation source. Figure 13 and Figure 14 reveal the processing procedure for a single signal and multiple signals, respectively. It must be pointed out that the surface wave can be removed in frequency-wavenumber domain if the wave packets are overlapped in the time-domain.

#### 4.2.2. Experimental Results

**Figure 15:**
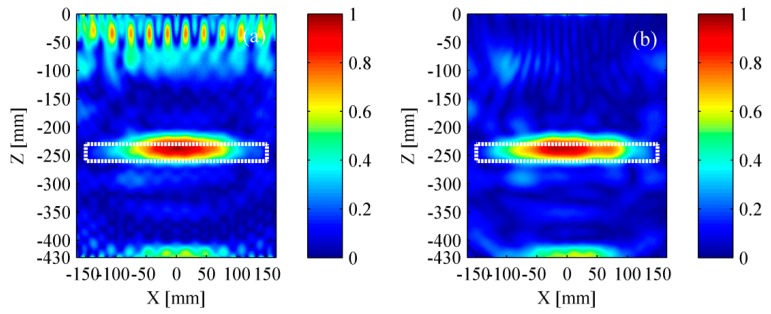
Experimental results based on the TFM. (**a**) Result with surface wave and (**b**) result without surface wave. The rectangle with white dotted line represents the location of a delamination.

**Figure 16:**
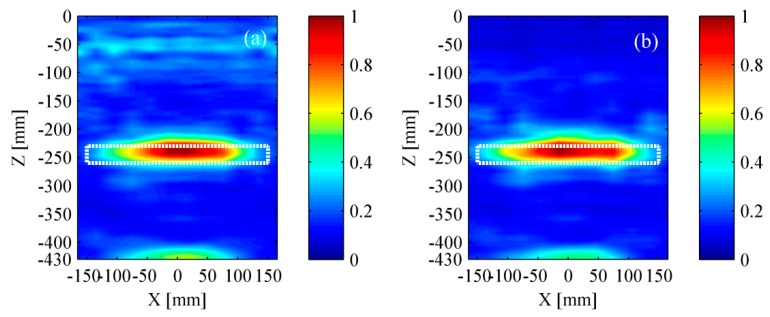
Experimental results based on the wavenumber method. (**a**) Result with surface wave and (**b**) result without surface wave. The rectangle with white dotted line represents the location of a delamination.

Figure 15 and Figure 16 show the experimental results based on TFM and the wavenumber method, respectively. The experimental results were in good agreement with the simulation results. It can be found that a relatively high accuracy was achieved if the surface wave was removed successfully. Meanwhile, it is reconfirmed that the wavenumber method can achieve a better lateral resolution.

## 5. Discussion

The TFM and wavenumber method have been used for characterizing the delamination in a ballastless slab track. Two methods are compared in the computational performance, lateral resolution, and longitudinal resolution. In addition, the difference between simulation and experimental results has been pointed out. Moreover, the delamination and the bottom of bed plate are compared in the imaging results. Finally, the influence caused by the amount of transducers has been analyzed. 

(1) The TFM and the wavenumber method are compared in the computational performance.

The wavenumber method can provide with the superior computational performance. We consider a linear array with J transducers. Each signal is recorded by *K* time samples. The output image is assumed with M × N pixels. The computational performances for the TFM and the wavenumber method can be evaluated by [31,32]:

(18) $$C_{TFM}=O(MNJ^{2})$$

(19) $$C_{\omega -k}=O(J^{2}K\log_{2}(J^{2}K)+MNJ+MN\log_{2}(MN))$$

where, the Landau notation O(⋅) is used to denote the order of computational complexity. $C_{TFM}$ and $C_{\omega -k}$ denote the computational complexities of TFM and wavenumber method, respectively. In this paper, the relevant parameters were set as: M = 490, N = 330, J = 12, and K = 2048. Therefore, the computational complexity ratio of TFM to wavenumber method was $C_{TFM}/C_{\omega -k}=2.306$ theoretically. In practice, the time cost of using TFM is given by $T_{TFM}=4.192$ s. While the time cost of using the wave number method is given by $T_{\omega -k}=2.011$ s. The times were measured on a personal computer with Core i7 3.40 GHz CPU and 24 GB RAM. It is easy to obtain the ratio $T_{TFM}/T_{\omega -k}\approx 2.084$. The theoretical and experimental results confirm that the wavenumber method can provide with the superior computational performance. Especially, the detection is implemented in a large area or the array consists of many transducers.

(2) The TFM and the wavenumber method are compared in the lateral resolution and the longitudinal resolution.

**Figure 17:**
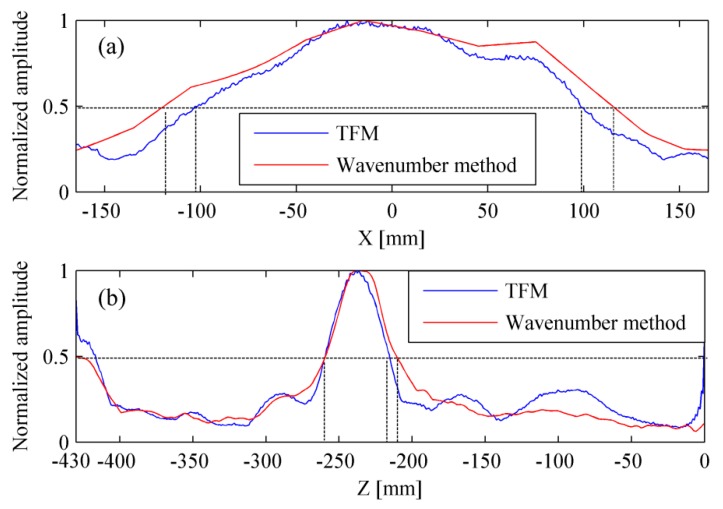
Profile curves of experimental results. (**a**) Lateral profile curve and (**b**) longitudinal profile curve.

In this paper, the actual length and thickness of the delamination were 300 mm and 30 mm, respectively. Figure 17 shows the profile curves of experimental results. The size of delamination is estimated by setting a threshold at the level of −6 dB and results are shown in Table 3. The measured delamination length is only 201 mm by using the TFM. However, this value reached up to 234 mm by using the wavenumber method. The measured delamination thickness is 45 mm by using the TFM, and this value was 49 mm by using the wavenumber method. 

It is easy to draw a conclusion that the wavenumber method is better than the TFM in the lateral resolution, but there is no significant difference in the longitudinal resolution. One reasonable explanation is that a mathematically rigorous solution to the inverse problem is obtained in wavenumber method, while the TFM only adopts the delay-and-sum beam-forming. Therefore, the wavenumber method can achieve better accuracy. It must be pointed out that the wavenumber method requires a linear array, while the TFM is applicable with a flexible transducer array.

(3) The difference between simulation results and experimental results is analyzed.

**Figure 18:**
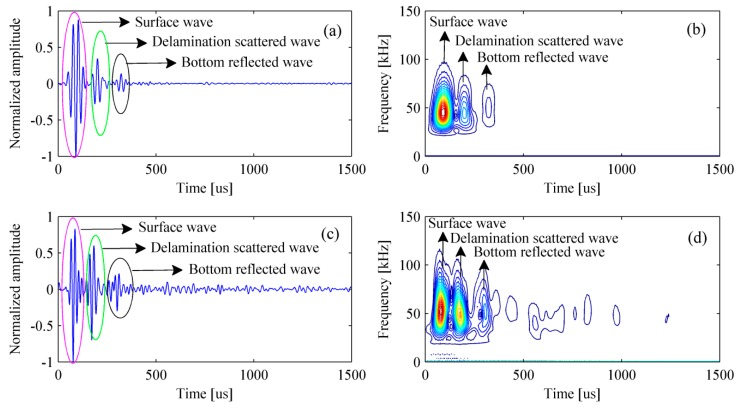
Comparison between the simulated signal and the experimental signal. (**a**) Simulated signal in time domain; (**b**) simulated signal in time-frequency domain; (**c**) experimental signal in time domain; and (**d**) experimental signal in time-frequency domain.

Experimental results show that the delamination and the bottom of bed plate can be easily identified without removing the surface wave, while they are obscure in simulation results. This discovery is obtained by comparing Figure 9a with Figure 15a, or comparing Figure 10a with Figure 16a. To explain this phenomenon, the simulated signal and the experimental signal were compared in time-domain. Figure 18a shows that the amplitude of surface wave is much higher than that of the delamination scattered wave and the bottom reflected wave. However, Figure 18c shows that their difference in amplitude is not so serious. Figure 18b,d is the time-frequency spectra for the simulated signal and experimental signal, respectively. The time-frequency spectra have further confirmed that the pseudomorphism caused by the surface wave is serious under the simulation condition.

(4) The location of the delamination is precise while the bottom of bed plate can not be identified very well.

On the one hand, it is not appropriate to assume the propagation speed as a constant during the process of implementing algorithms. In fact, the ballastless slab track is a typical multi-layers structure. We should pay more attention to the fact that the wave propagation velocities in multi-layers are different. More specifically, the shear wave velocity is about 2466 m/s in the slab track. This value is about 2377 m/s in the self-compacting concrete layer and the bed plate. Although the difference in velocity is not obvious, however, it still can affect the test quality at the bottom of bed plate. 

On the other hand, the imagery quality is relevant to the attenuation coefficient of materials. In other words, the amplitude of the scattered wave caused by the delamination is higher than that of the reflected wave from the bottom because of the attenuation. Consequently, the delamination is conspicuous while the bottom of bed plate is obscure.

(5) The amount of transducers has a great influence on the test results.

**Figure 19:**
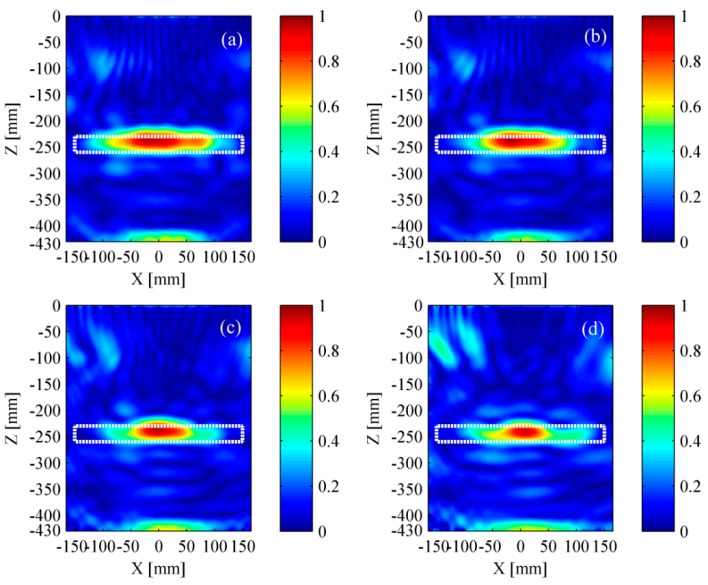
Experimental results based on the TFM. (**a**) Twelve transducers; (**b**) ten transducers; (**c**) eight transducers; and (**d**) six transducers. The rectangle with white dotted line represents the location of a delamination.

**Figure 20:**
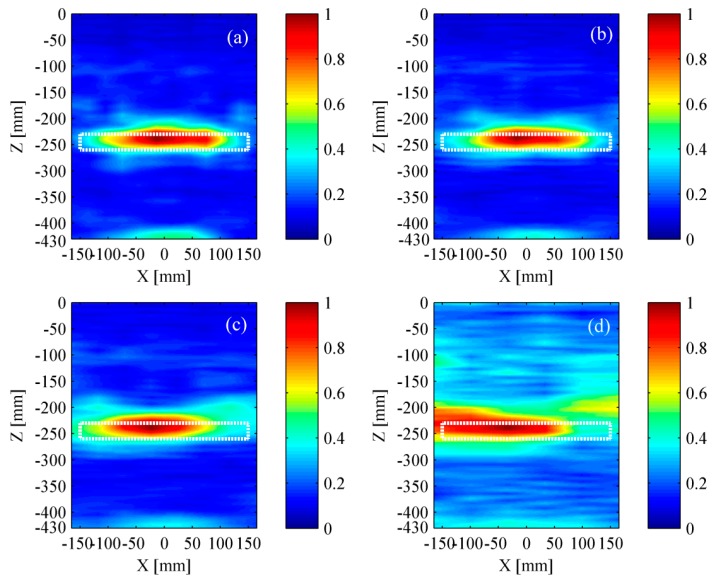
Experimental results based on the wavenumber method. (**a**) Twelve transducers; (**b**) ten transducers; (**c**) eight transducers; and (**d**) six transducers. The rectangle with white dotted line represents the location of a delamination.

In order to make the result be more intuitive, all the analysis was based on the experimental signals, which have been removed surface waves. The test results by applying different amount of transducers are shown in Figure 19 and Figure 20. It could be found that the identified delamination length was gradually shortening with the decrease of transducers. Meanwhile, the expansion of the residual pseudomorphism was inevitable. Generally, the more the transducers are, the better test results can be achieved. 

## 6. Conclusions

The paper presents the TFM and wavenumber method for the delamination localization in ballastless slab track. Both simulation results and experimental results demonstrate that the delamination and the bottom of the bed plate can be identified by the proposed methods. The conclusions can be drawn as follows:

(1) The test result has been distinctly improved as the near-surface pseudomorphism is restrained by applying the technique of removing the surface wave.
(2) Compared to TFM, the wavenumber method can provide with the better computational performance and the lateral resolution. However, they have no significant difference in the longitudinal resolution.
(3) It can be observed that the delamination is conspicuous while the bottom of bed plate is obscure in the image. The explanation could be the inhomogeneous propagation speed and the attenuation existed in concrete.
(4) The test result is relevant to the amount of transducers. Generally, the more the transducers are, the better test result can be achieved.
(5) The low frequency air-coupled ultrasonic inspection combined with Lamb wave is viewed as a preliminary step for obtaining the location of the delamination in the transversal direction.
(6) Compared with the classical delay-and sum (DAS) method, the wavenumber method can greatly reduce the computational time while retaining a relatively favorable accuracy.

The proposed methods can provide useful information for the early warning of the structural failure. It must be noted that the accurate shear-wave velocity and removing surface wave are very crucial for the delamination detection. 

## 7. Future Work

Detection with a large area will be taken into consideration for future studies. In practice, the delamination location is usually unknown in a ballastless slab track. For this reason, a large amount of data must be processed if the proposed methods are employed for the detection with a large area. Naturally, consuming much time is inevitable. In addition, it is not flexible, as the DPC transducers still have to contact the surface of the detected object. Fortunately, air-coupled ultrasonic inspection combined with Lamb wave is a promising non-contact method for the rapid and convenient inspection [42,43,44]. More specifically, the low frequency air-coupled ultrasonic inspection combined with Lamb wave is viewed as a preliminary step for obtaining the location of the delamination in the transversal direction. On this basis, the proposed methods are implemented on the small scanning region where delamination is likely to exist. Consequently, delamination can be localized both in the transversal and longitudinal directions. The detailed solutions will be introduced in future work.

## Figures and Tables

**Table 1:** Parameters of the finite element model.

Property	Slab Track	Self-Compacting Concrete Layer	Bed Plate	Subgrade
Concrete strength	C60	C40	C40	/
Density (kg/m^3^)	2500	2500	2500	1850
Elasticity modulus (Gpa)	36.5	32.5	32.5	25
Poisson’s ratio	0.2	0.2	0.2	0.35
Thickness (mm)	200	60	170	200
Velocity of shear wave	2466	2377	2377	2237

**Table 2:** Parameters of the ultrasonic transducer array.

Property	Value
Amount of transducers	12
Central distance between adjacent transducers (mm)	30
Central frequency of the excitation signal (kHz)	50
Bandwidth by the level −6 dB (kHz)	20–80

**Table 3:** The size of delamination evaluated by TFM and wavenumber method.

Method	Delamination Length (mm)	Delamination Thickness (mm)
TFM	201	45
Wavenumber method	234	49
